# Location determination of metal nanoparticles relative to a metal-organic framework

**DOI:** 10.1038/s41467-019-11449-6

**Published:** 2019-08-01

**Authors:** Yu-Zhen Chen, Bingchuan Gu, Takeyuki Uchida, Jiandang Liu, Xianchun Liu, Bang-Jiao Ye, Qiang Xu, Hai-Long Jiang

**Affiliations:** 1 Hefei National Laboratory for Physical Sciences at the Microscale, CAS Key Laboratory of Soft Matter Chemistry, Department of Chemistry, Collaborative Innovation Center of Suzhou Nano Science and Technology, University of Science and Technology of China, Hefei, Anhui 230026 P. R. China; 2 College of Chemistry and Chemical Engineering, Qingdao University, Qingdao, Shandong 266071 P. R. China; 3 State Key Laboratory of Particle Detection and Electronics, University of Science and Technology of China, Hefei, Anhui 230026 P. R. China; 4 National Institute of Advanced Industrial Science and Technology (AIST), Ikeda, Osaka 563-8577 Japan; 5 State Key Laboratory of Catalysis, Dalian Institute of Chemical Physics, Chinese Academy of Sciences, Zhongshan Road 457, Dalian, 116023 P. R. China

**Keywords:** Metal-organic frameworks, Metal-organic frameworks, Heterogeneous catalysis, Catalyst synthesis

## Abstract

Metal nanoparticles (NPs) stabilized by metal-organic frameworks (MOFs) have been intensively studied in recent decades, while investigations on the location of guest metal NPs relative to host MOF particles remain challenging and very rare. In this work, we have developed several characterization techniques, including high-angle annular dark-field scanning transmission electron microscopy (HAADF-STEM) tomography, hyperpolarized ^129^Xe NMR spectroscopy and positron annihilation spectroscopy (PAS), which are able to determine the specific location of metal NPs relative to the MOF particle. The fine PdCu NPs confined inside MIL-101 exhibit excellent catalytic activity, absolute selectivity and satisfied recyclability in the aerobic oxidation of benzyl alcohol in pure water. As far as we know, the determination for the location of metal NPs relative to MOF particles and pore structure information of metal NPs/MOF composites by ^129^Xe NMR and PAS techniques has not yet been reported.

## Abstract (web summary)

While metal nanoparticles (NPs) stabilized by metal-organic frameworks (MOFs) have been intensively studied, the determination of the location of guest metal NPs relative to host MOF particles remains challenging. Here the authors develop several techniques to determine the specific location of metal NPs relative to the MOF particles.

## Introduction

Noble metal nanoparticles (NPs) have attracted intensive interest due to their particular electronic structure and physiochemical properties as well as potential applications in many fields, especially in catalysis^1–7^. However, the limited storage and the high cost of noble metal, such as Pt, evoke great efforts to develop their alternative catalysts. The combination of a transition metal with noble metal to form bimetallic NPs in an alloy or core-shell structure has become an effective strategy not only in decreasing the consumption of noble metals but also largely improving the catalytic performance because of synergistic effect between two metals^8–14^. For instance, the integration of Pt with Cu into bimetallic PtCu alloy NPs exhibits enhanced performance of Pt in electrooxidation of different alcohols^12^. In addition, the well-known size effect of metal nanoparticles (NPs) strongly affects their catalytic performance: small MNPs with large surface area are highly desirable. Unfortunately, these small MNPs with high surface energies readily aggregate to form larger MNPs with deteriorated catalytic activity^15–17^. Therefore, tremendous efforts have been dedicated to the exploitation of effective methods to stabilize MNPs, among which porous materials have been demonstrated to be very useful to restrict the growth of MNPs^18–23^.

Metal–organic frameworks (MOFs), emerging as a unique class of porous materials, have captured great research interest due to their highly ordered and tailorable pore structure, superhigh surface area, and potential applications in many fields, such as gas sorption and separation, sensor, drug delivery, etc.^24–33^. The permanent porosity of MOFs offers inherent conditions to confine small MNPs, and the crystalline porous structure of MOFs is expected to prevent the migration and aggregation of MNPs^34–48^. There have been quite a few reports on the stabilization of MNPs by MOFs based on different synthetic strategies, which can be mainly divided into three main approaches: (1) synthesis of surfactant-protected MNPs, followed by MOF epitaxial growth to give MNPs@MOF;^34–36^ (2) the assembly of surfactant-protected MNPs and MOF to give MNPs/MOF;^37–39^ (3) introduction of metal precursors into MOFs and subsequent reduction in situ to afford MNPs@MOF^40–48^. Among them, MNPs are well encapsulated inside and supported on MOFs, respectively, obtained by the first and second approaches, while the surfactant-encapsulated MNPs in obtained nanocomposites are actually unfavorable as the surfactant possibly blocks the active sites to some extent. Delightedly, MNPs are surfactant free in MNPs/MOF obtained by the last synthetic approach, and they exhibit considerable catalytic performances due to the fully accessible and clean surface of MNPs. Unfortunately, it remains a grand challenge to glean experimental evidences whether MNPs stay on or inside MOF particles.

Currently, the assertion for MNP location relative to MOF particles are mostly based on the sizes between MNPs and MOF pores, where smaller sizes of MNPs than MOF cavities are deemed to be embedded inside MOFs. Recently, size-selective catalysis has been employed as an important evidence to distinguish whether or not MNPs are encapsulated into MOF pores: small substrate has high conversion while large substrate cannot be accessible to MNPs inside a MOF thus not be reacted^34–36^. Although this solution is facile and the judgment is usually correct, it does not definitely reflect the real situation as the low catalytic efficiency of large substrates is sometimes ascribed to their inferior reaction kinetics to small ones. To provide credible information on the location of MNPs relative to MOF particles, we conducted X-ray absorption spectroscopy (XAS) to clearly demonstrate the incorporation of ultrafine Au_2.5_ clusters in a MOF with fluoro-coated channels^42^. However, synchrotron radiation source is not easily available, and MNPs are usually much larger than the clusters with several atoms only, even they are incorporated inside MOFs. XAS might not be the first choice for the location determination of MNPs relative to MOF particles. Therefore, to better understand the microstructure and structure–activity relationship of MNPs/MOF composites, it is highly desired to develop not only reliable but also readily available characterization techniques to determine the location of MNPs relative to MOF particles.

The ^129^Xe NMR spectroscopy is specifically sensitive to the void spaces. In the past decades, it has become a well-established tool to investigate the pore structure information of porous materials, such as zeolite, porous carbon, porous polymer, mesoporous silica, etc.^49–53^, and complementary to conventional techniques that describe the solid part of materials. Accordingly, Xenon is also a sensitive probe to characterize the pores in MOFs by using either thermally polarized or hyperpolarized xenon^54–57^, although it has never been applied to the MNPs/MOF system yet thus far. Meanwhile, positron annihilation spectrometry (PAS) is one of the most powerful tools to detect vacancy-type defects, open volumes, and pores by measuring the lifetime of the positron^58–61^. The nondestructive and auto detective property makes it an effective method to study metals, semiconductors, and other porous materials^60^, Therefore, PAS should be also suitable to characterize the location of MNPs relative to MOF particle.

In this work, we have successfully incorporated monometallic Pt and bimetallic PtCu NPs into a MOF, with Pt and PtCu NP sizes of 1.5 nm and 1.7 nm, respectively, via a double-solvent approach (DSA). Three advanced techniques, namely transmission electron microscopy (TEM) tomography, ^129^Xe NMR spectroscopy, as well as PAS, have been cooperatively investigated the location of Pt and PtCu NPs relative to the host MOF particles. To the best of our knowledge, both ^129^Xe NMR and positron annihilation techniques have not yet been employed to provide decisive evidences on whether MNPs are confined inside or located on MOFs. Significantly, thanks to the ultrafine PtCu NPs, the resultant PtCu@MOF exhibits excellent catalytic activity, selectivity, and recyclability toward the aerobic oxidation of benzyl alcohol in pure water.

## Results

### Synthesis and characterization of PtCu@MIL-101

The representative mesoporous MOF, Cr-MIL-101 with a molecular formula of Cr_3_X(H_2_O)_2_O(BDC)_3_·nH_2_O (BDC = benzene-1,4-dicarboxylate, X = F or OH, *n* ≈25)^62^, was chosen as a host matrix to encapsulate MNPs due to its large specific surface area (BET, >3600 m^2^ g^−1^), high-chemical stability, appropriate pores (2.9 and 3.4 nm) accessible through two pore windows of *ca*. 1.2 and 1.6 nm, respectively. The giant cages in MIL-101 are hydrophilic, which allow us to adopt a DSA approach to rationally incorporate the metal precursors into the cavities of MIL-101^43,45^. In brief, the precursor solution involving H_2_PtCl_6_·6H_2_O and/or Cu(NO_3_)_2_·3H_2_O with a volume slightly less than the MOF pore volume was absorbed into the pores of dehydrated MIL-101, thanks to the capillary force and hydrophilic interaction. Subsequently, the resultant metal precusors @MIL-101 composite was reduced by 20% H_2_/Ar to afford tiny Pd and/or PdCu NPs (typically, 0.5 wt% metal loading otherwise mentioned) encapsulated in MIL-101.

No diffraction peak in the powder X-ray diffraction (PXRD) profiles can be assigned to Pt and PtCu NPs in the resultant nanocomposites (Supplementary Fig. 1), revealing that the obtained MNPs could be very small and/or low content of MNPs. The BET surface areas of as-synthesized MIL-101, Pt@MIL-101, Pt_2_Cu_1_@MIL-101, Pt_1_Cu_1_@MIL-101, Pt_1_Cu_2_@MIL-101, Pt_1_Cu_3_@MIL-101, Pt_1_Cu_4_@MIL-101, and Cu@MIL-101 are 2904, 2645, 2545, 2564, 2329, 2302, 2254, and 2479 m^2^ g^−1^, respectively, implying that the cavities of MOF are possibly occupied by highly dispersed MNPs (Supplementary Fig. 2). Inductively coupled plasma atomic emission spectrometry (ICP-AES) for several representative samples has confirmed that the actual contents of Pt and Cu are close to the nominal values, and the Pt/Cu ratios almost agree the predesigned trend (Supplementary Table 1). An obvious positive shift in the binding energy of Cu 2*p*_3/2_ can be observed for PtCu@MOF in reference to that of Cu@MOF from the X-ray photoelectron spectroscopy (XPS), which might be due to that the electrons on Cu surface flow into Pt considering the higher Fermi level of Cu than Pt (Supplementary Fig. 3)^9^.

**Fig. 1:**
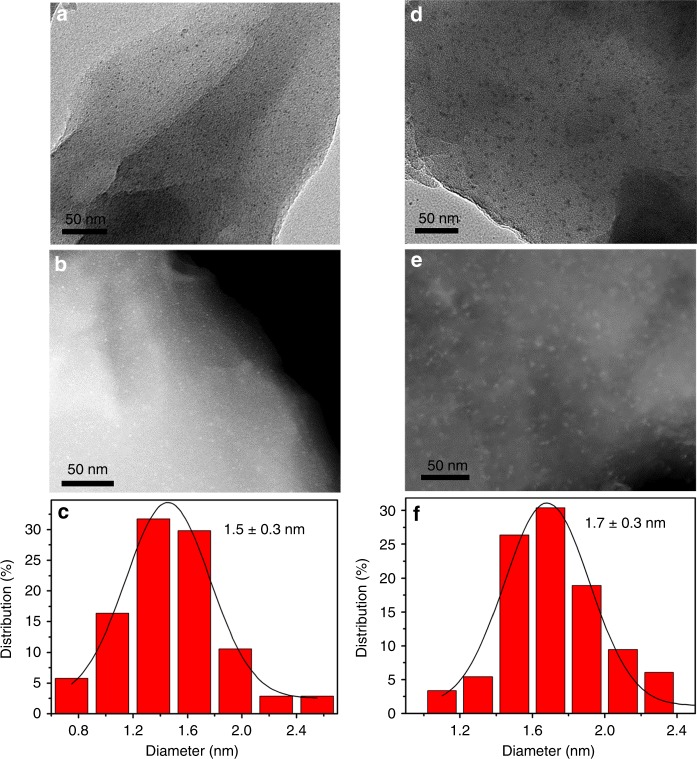
TEM, HAADF-STEM images and size distribution. **a** TEM, **b** HAADF-STEM images, and **c** the corresponding size distribution of Pt NPs in Pt@MIL-101. **d** TEM, **e** HAADF-STEM images, and **f** the corresponding size distribution of PtCu NPs in Pt_1_Cu_2_@MIL-101

**Fig. 2:**
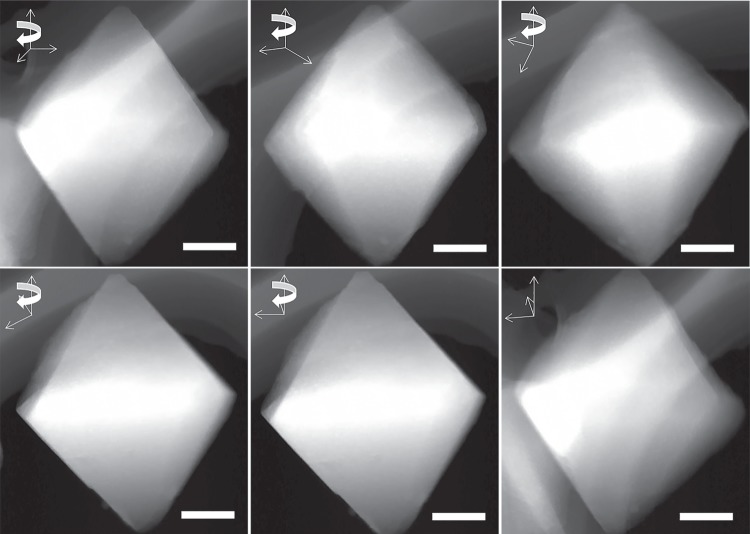
HAADF-STEM images. The representative HAADF-STEM images captured from the video with a series of tilting angles for PtCu@MIL-101 sample taken with 2° tilt increment step from −62.6° to 62.6°. Alpha tilt axis is parallel with Z direction shown on the images. The scale bar on the images is 100 nm

The TEM and high-angle annular dark-field scanning transmission electron microscopy (HAADF-STEM) images for Pt@MIL-101 (Fig. 1a, b) and PtCu@MIL-101 (Fig. 1d, e, Pt_1_Cu_2_@MIL-101 as a representative hereafter unless otherwise stated) show Pt and PtCu NPs in ultrafine sizes of ~1.5 and ~1.7 nm (Fig. 1c, f), respectively. As far as we know, this is among the smallest sizes of bimetallic NPs in the presence of surfactant protection thus far^45^. The smaller sizes of MNPs than the MOF pores hint the possible encapsulation of MNPs inside the giant cages. To confirm this, HAADF-STEM tomography was employed to examine the intrinsic morphology and spatial distribution of the nanocomposites^37^. The tomographic data were reconstructed to provide more detailed information for the 3D imaging of the real structure. A series of 3D HAADF-STEM images were taken for Pt@MIL-101 (Supplementary Fig. 4) and Pt_1_Cu_2_@MIL-101 (Fig. 2) at consecutive tilt angles from −62.6° to 62.6° with each 2° tilt increment (videos 1 and 2 in the Supplementary Information). No agglomerated large Pt or PtCu NPs can be observed during the tilting process, revealing that MNPs are highly dispersed and embedded inside the MOF pores.

**Fig. 3:**
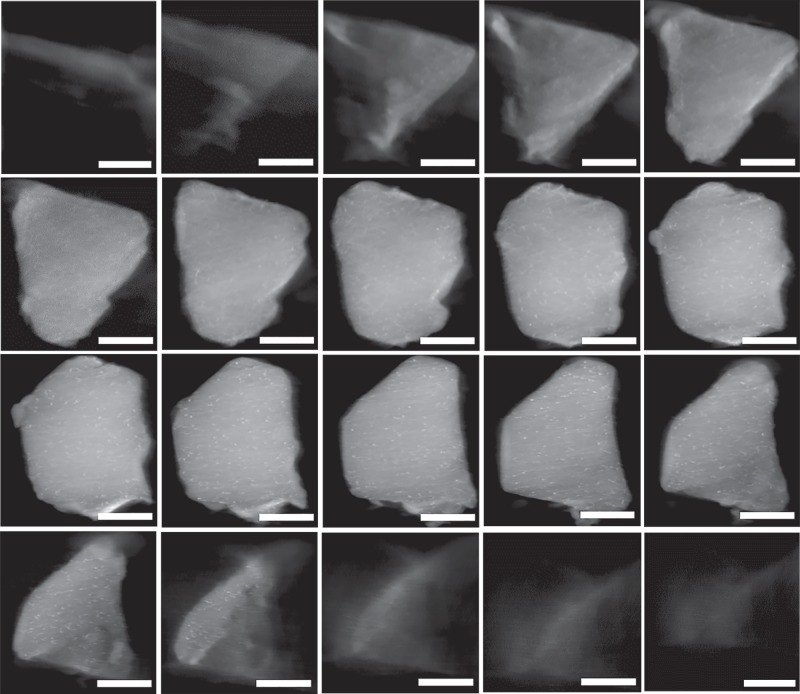
Reconstructed slice images. Selected reconstructed slice images (observed in the order of left to right and top to down) throughout the entire Pt_1_Cu_2_@MIL-101 skeleton. The scale bar on the images is roughly ~200 nm

To further prove this, a series of tomographic slices unambiguously present the highly dispersed ultrafine PtCu NPs throughout the whole MIL-101 skeleton, demonstrating that almost all PtCu NPs are indeed trapped inside the MOF pores (Fig. 3, and video 3 in the Supplementary Information). The similar results gleaned from the tomographic slices of Pt@MIL-101 also confirm the encapsulation of Pt NPs inside MIL-101 (Supplementary Fig. 5 and video 4 in the Supplementary Information). The above results clearly suggest the successful incorporation of Pt^2+^/Cu^2+^ into MOF cages via the strategy of DSA and the ideal confinement effect of MIL-101 for Pt and PtCu NPs.

**Fig. 4:**
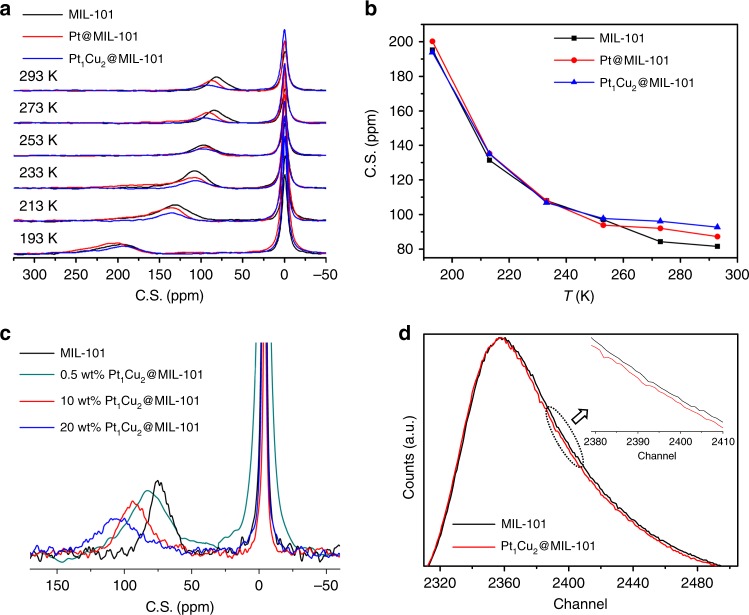
^129^Xe NMR and positron lifetime characterizations. **a** Temperature-dependent hyperpolarized ^129^Xe NMR spectra for MIL-101, Pt@MIL-101, and PtCu@MIL-101. The measurements were performed in the range of 193–293 K. **b** The ^129^Xe chemical shift difference for MIL-101, Pt@MIL-101, and Pt_1_Cu_2_@MIL-101 at different temperatures. **c** Hyperpolarized ^129^Xe NMR spectra performed at 293 K for MIL-101, and Pt_1_Cu_2_@MIL-101 with various metal percentages. **d** Positron lifetime spectra for MIL-101 and 0.5 wt% Pt_1_Cu_2_@MIL-101 based on positron annihilation experiments

Encouraged by the above results, hyperpolarized ^129^Xe NMR technique was applied to illustrate the location of MNPs relative to MIL-101 particles. ^129^Xe, a noninvasive gas molecule, is able to mimic the behavior of guest atoms/molecules inside the pores, as the high polarizability of xenon electron cloud make it very sensitive to physical interactions with guest species in the pores, resulting in ^129^Xe NMR chemical shift variation in reference to pristine porous materials (with empty pores). Therefore, the variable-temperature ^129^Xe NMR spectroscopy has been recorded by using a continuous flow of hyperpolarized xenon adsorbed into MIL-101, Pt@MIL-101 and PtCu@MIL-101. Given that ^129^Xe is chemically inert with a diameter of 0.44 nm, much smaller than the pore windows and diameters of MIL-101, or even MIL-101 loaded with MNPs, it would easily go through the pores to access the guest MNPs. Prior to ^129^Xe NMR measurement, the samples were evacuated to remove residual guest molecules. Temperature-dependent ^129^Xe NMR spectra for MIL-101, Pt@MIL-101, and PtCu@MIL-101 are shown in Fig. 4a. The peaks at 0 ppm are from the free ^129^Xe gas, and the signals at lower field are originated from the absorbed Xe molecules inside MIL-101 pores. The signal intensity difference of the ^129^Xe NMR signals for MIL-101, Pt@MIL-101, and PtCu@MIL-101 at a fixed temperature might be also related to the pore size and the stronger Xe adsorption at low temperatures. The chemical shifts of Xe molecules absorbed inside MIL-101 pores increase from ~85 to ~200 ppm along with decreased temperature from 293 to 193 K for all samples. This is a common trend in variable-temperature ^129^Xe NMR spectra, mainly owing to the extended residence time of Xe on the internal surfaces and increased Xe–Xe interactions at lower temperatures. At a fixed temperature (e.g., 293 K) close to room temperature, the chemical shift (value) in hyperpolarized ^129^Xe NMR spectra increases in the order of MIL-101 < Pt@MIL-101 < PtCu@MIL-101 due to their different sizes of the cavities (Fig. 4b). Given that the lower Xe concentration brings the greater chemical shift toward downfield, the data clearly suggest the cavity sizes in the order of MIL-101 > Pt@MIL-101 > PtCu@MIL-101, in accordance with the occupied MOF pores by Pt and PtCu NPs, with slightly larger sizes of PtCu than Pt.

While some difference of chemical shift of ^129^Xe adsorbed in the three samples can be observed at high temperature (close to room temperature), they are indeed similar at low temperatures (Fig. 4a, b). The result is mainly because of the very low metal loading (<1 wt%) and small sizes of metal NPs in the composites, where only very limited MOF cavities are occupied. Moreover, along with decreasing temperatures, the higher Xe concentration in MIL-101 leads to enhanced interaction between Xe–Xe, in comparison with MNPs@MOF samples, which brings the greater chemical shift toward low field. This leads to the resultant close chemical shift in all three samples at low temperatures (Fig. 4a).

To further verify the applicability of ^129^Xe NMR to identify the location of MNPs and augment the difference of chemical shift among these samples, 10 wt% Pt@MIL-101, and 10 and 20 wt% PtCu@MIL-101 have been deliberately synthesized for ^129^Xe NMR measurement (Supplementary Fig. 6a, b). As expected, the chemical shift of ^129^Xe adsorbed shows distinct difference and the chemical shift increases in the order of MIL-101 <10 wt% Pt@MIL-101 <10 wt% PtCu@MIL-101 due to their significantly different pore sizes. In addition, at a fixed temperature (e.g., 293 K), hyperpolarized ^129^Xe NMR spectra for MIL-101, and PtCu@MIL-101 with various PtCu NPs quality (0.5, 10, and 20 wt%, n_Pt_/n_Cu_ = 1/2) show that the Xe chemical shifts gradually increase along with the increased metal percentage from 0.5 wt% to 20 wt% of PtCu NPs in PtCu@MIL-101 (Fig. 4c). The results further demonstrate that the MNPs are mainly encapsulated in MOF pores.

To further verify the above results, positron annihilation (PA) technique has been adopted to evaluate the location of MNPs relative to the MOF particles, as positrons are very sensitive to the pores and/or defects on the atomic scale, which act as trap centers for positron. The positron lifetime spectra measured in nitrogen atmosphere (1 atm) with three lifetime components, τ1(I1), τ2(I2), and τ3(I3), by using LTv9 program^63^ for MIL-101 and 0.5 wt% PtCu@MIL-101 samples are shown in Fig. 4d. Their similar lifetime components are mainly due to the very low metal loading (<1 wt%) and tiny MNPs in the sample. Therefore, 10 and 20 wt% Pt@MIL-101 as well as 10 and 20 wt% PtCu@MIL-101 have been deliberately synthesized for PA measurement (Supplementary Fig. 6c, d, and Table 1). The lifetimes (τ1, τ2, and τ3) are similar for three samples, while the intensities are quite different after loading Pt or PtCu NPs inside the MOF pores, especially for PtCu@MIL-101. Lifetimes shorter than 200 ps are usually found in the materials with high electron density, such as metal, alloy, or some of semiconductors^64^. Given that the electron density of MOF is relatively low, the positronium (Ps) is prone to form in the open volume regions. Therefore, the τ1 is most likely originated from the average of PA with free electrons and the spin singlet (p-Ps, mean lifetime of 125 ps) annihilation. The second lifetime (τ2) component at around 370 ps stems from the PA with the electrons in pores or defects. The intensities (I1, I2, and I3) for all samples are shown in Table 1 and Supplementary Fig. 7. The predominant intensity I2 (more than 70%) implies that most of the positrons in the sample are trapped by the pores and/or defects in MOF. The longest lifetime of component τ3 longer than 2 ns represents the remained triplet states (o-Ps) formed at the surface or the interspace among the MOF particles. However, the intensity I3 is quite low (less than 1.5%), which is probably because the o-Ps are quenched through pick-off or the other interactions due to the nitrogen molecules in the system. From the intensity comparison of three samples, after loading of 10 wt% PtCu NPs, I2 decreases from 88.6 to 76.4%, and I1 increases from 10.9 to 22.1%, which suggests that after loading of PtCu NPs, the free space in MOF pores becomes smaller and the long lifetime component decreases. Meanwhile, the PtCu NPs in MIL-101 might make o-Ps more easily quenched and quickly annihilated, which also leads to the increase of I1 and decrease of I2. In summary, the unchanged τ indicates that the main structure and the type of defects and pores (sensed by positrons) are remained before and after the NPs loading. However, the increase of I1 and decrease of I2 adequately demonstrates the incorporation of Pt or PtCu NPs inside the MOF pores. This can be explained by the obvious difference of the lifetime component for MIL-101, 10 wt% Pt@MIL-101, and 10 wt% PtCu@MIL-101 from the raw data (Supplementary Fig. 6c, d). There are quite a few possible reasons for the visible difference of I3 or τ3 between MIL-101 and 10 wt% PtCu@MOF (Table 1). Besides the MOF material itself, as no regular changes between tau3 and I3 observed in previous reports^64,65^, the existence of Cu element would be responsible for the reduced lifetime. It is reported that the positrons prefer to annihilate on the Cu surface due to the lower positron affinity of Cu^66,67^. Moreover, the origin of the component (τ3) is complicated: the o-Ps can be formed at the surface, or the interior, or even the interface of the sample, and surface morphology change and some uncertain factors such as background noise may also influence the o-Ps formation. As a control, the PA spectrum of an as-synthesized MOF-5 has also been measured at room temperature in nitrogen atmosphere. The significant difference of the long-lifetime component between MIL-101 and MOF-5 can be obtained from the results (Table 1; Supplementary Fig. 8); moreover, the third component intensity (I3, 0.54%) of MIL-101 is much lower than that (around 11%) of MOF-5. These results suggest that the PAS are highly dependent on the specific MOF, and different MOF structures can present distinctly different results^68–70^.

**Table 1:** Positron lifetime parameters

Sample	τ_1_ (ps)	τ_2_ (ps)	τ_3_ (ns)	I_1_ (%)	I_2_ (%)	I_3_ (%)
MIL-101	192.0 ± 1.5	365.9 ± 3.3	2.65 ± 0.20	10.9 ± 1.2	88.6 ± 2.0	0.54 ± 0.04
0.5 wt% PtCu@MOF	188.0 ± 2.4	368.5 ± 1.6	2.59 ± 0.12	12.0 ± 0.8	86.4 ± 1.8	0.65 ± 0.03
10 wt% Pt@MOF	180.1 ± 9.6	371.9 ± 2.5	2.45 ± 0.16	14.7 ± 1.3	84.5 ± 1.3	0.78 ± 0.05
10 wt% PtCu@MOF	184.3 ± 5.8	374.5 ± 1.7	2.10 ± 0.04	22.1 ± 0.8	76.4 ± 0.8	1.49 ± 0.03
20 wt% Pt@MOF	184.1 ± 6.0	373.0 ± 2.5	2.34 ± 0.07	23.4 ± 0.5	78.9 ± 1.0	1.06 ± 0.05
20 wt% PtCu@MOF	186.0 ± 5.8	377.4 ± 1.1	2.05 ± 0.06	25.3 ± 0.4	73.4 ± 1.2	1.56 ± 0.02
MOF-5	215.8 ± 6.3	431.9 ± 5.7	2.90 ± 0.19	30.7 ± 1.5	58.2 ± 1.4	11.09 ± 0.3

The τ_1_, τ_2_, and τ_3_ represent lifetime components, I_1_, I_2_, and I_3_ represent the intensities. These results were measured at nitrogen atmosphere (1 atm)

### Catalytic oxidation of alcohols by PtCu@MIL-101 catalysts

**Fig. 5:**
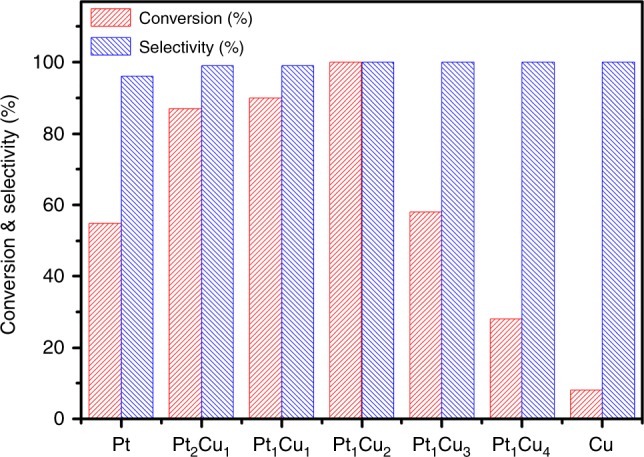
Oxidation of benzyl alcohol over PtCu@MIL-101 catalysts. Conversion and selectivity of catalytic oxidation of benzyl alcohol over PtCu@MIL-101 catalysts with different Pt/Cu molar ratios. Reaction conditions: catalyst (100 mg), alcohol (0.2 mmol), H_2_O (5 mL), O_2_ (0.5 MPa), 373 K, reaction time for each run is 5 h

Encouraged by the perfect encapsulation of ultrafine Pt and PtCu NPs inside MIL-101, the selective aerobic oxidation of primary alcohols with molecular oxygen in water was investigated to evaluate the catalytic performance of the nanocomposites. Figure 5 summarizes the catalytic conversion and selectivity for the oxidation of benzyl alcohol over PtCu@MIL-101 catalysts with various Pt/Cu molar ratios. The results indicate that PtCu@MIL-101 exhibit superior catalytic performance to monometallic Pt@MIL-101 and Cu@MIL-101 counterparts due to the synergistic effect between Pt and Cu species in the bimetallic catalysts. Evidently, the optimized PtCu@MIL-101 catalyst with Pt/Cu molar ratio of 0.5 exhibits the highest  conversion (>99%) and selectivity (100%) among all the catalysts with different Pt/Cu ratios. To demonstrate the advantages of the confinement effect of MIL-101, Pt_1_Cu_2_/MIL-101 with PtCu NPs mainly located on the surface of MIL-101 prepared by incipient wetness impregnation for comparison, exhibits lower yield (85%) to the target product under similar conductions, possibly due to the aggregated PtCu NPs with the lack of sufficient confinement effect.

### Recycling test and oxidation of various aromatic alcohols

**Fig. 6:**
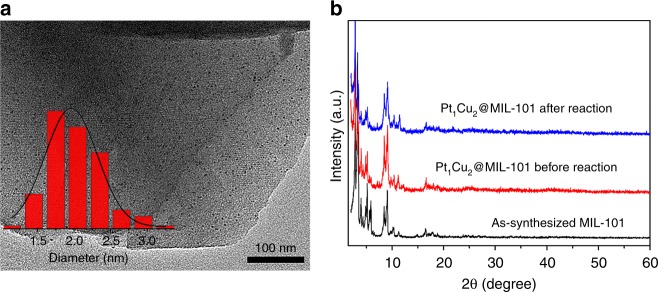
TEM image and PXRD patterns. **a** The TEM image and (inset) the corresponding size distribution of PtCu NPs in Pt_1_Cu_2_@MIL-101 after three catalytic cycles. **b** Wide-angle PXRD patterns of MIL-101 and Pt_1_Cu_2_@MIL-101 before and after catalytic reaction

No significant loss of crystallinity for the MOF and no identifiable peak for PtCu NPs can be observed in the PXRD pattern of PtCu@MIL-101 after reaction, indicating its high stability and good confinement of MIL-101 (Fig. 6b). Moreover, the catalytic activity and selectivity over Pt_1_Cu_2_@MIL-101 almost remains preserved during three cycles (Supplementary Fig. 9). No significant aggregation occurs to PtCu NPs even after three times of use from the representative TEM image for Pt_1_Cu_2_@MIL-101 (Fig. 6a). However, the contrast sample Pt_1_Cu_2_/MIL-101, with PtCu NPs supported on the external surface of the MOF, presents apparent aggregation of PtCu NPs from 3.1 to ~7.4 nm after three cycles under identical reaction conditions, causing the fading of recycling performance during stability test (Supplementary Figs. 10, 11). These results unambiguously suggest that the superb confinement effect offered by MIL-101 plays a vital role in preventing from aggregation and the excellent catalytic performance of tiny PtCu NPs, highlighting the importance of the MNP location relative to MOF particles. Encouraged by the outstanding catalytic performance of PtCu@MIL-101 in the oxidation of benzyl alcohol, we have extended this reaction to more substrates. To our delight, diverse substituted benzyl alcohols and heteroaromatic alcohols were completely oxidized within 5 h to corresponding aldehydes with almost absolute selectivity (Table 2), well demonstrating the general applicability of PtCu@MIL-101 catalysts. To further verify the good conversion and selectivity of the catalyst, the reaction with increased amount of substrate has been examined (Table 2, entry 12). The oxidation of alcohol can be almost completed with 93% conversion and 98% selectivity to benzaldehyde within 12 h, indicating the great adaptability of Pt_1_Cu_2_@MIL-101. Moreover, no detectable benzoic acid product even after extended reaction time indicates the excellent selectivity toward aldehyde by Pt_1_Cu_2_@MIL-101.

**Table 2:** Selective aerobic oxidation of various aromatic alcohols over Pt_1_Cu_2_@MIL-101^a^

^a^Reaction conditions: catalyst (100 mg), alcohol (0.2 mmol), H_2_O (5 mL), O_2_ (0.5 MPa), 373 K, reaction time is 5 h. ^b^Yield of aldehyde was analyzed by GC, and *n*-dodecane was used as the internal standard. ^c^Substrate (0.1 mmol). ^d^Substrate (0.5 mmol, reaction time is 12 h)

### Size-selective catalysis over Pt_1_Cu_2_@MIL-101 and Pt_1_Cu_2_/C

To further demonstrate the intact MIL-101 structure after loading MNPs, size-selective catalysis over MNPs@MIL-101 has been conducted. To this end, styrene (molecular size ~3.5 Å), cyclooctene (molecular size ~5.7 Å), and tetraphenyl ethylene (11.6 Å) were chosen for hydrogenation reactions (Supplementary Table 2). For comparison, we prepared the Pt_1_Cu_2_/graphite carbon (denoted Pt_1_Cu_2_/C) as a control, where PtCu NPs were located on the external surface of graphite carbon. Both Pt_1_Cu_2_@MIL-101 and Pt_1_Cu_2_/C showed >99% conversion of styrene after 10 min reaction at room temperature, indicating that MIL-101 shell almost has no influence on the diffusion of styrene. For cyclooctene with a larger molecular size, Pt_1_Cu_2_@MIL-101 gives 29 and 70% conversions while Pt_1_Cu_2_/C achieves 56 and >99% conversions, respectively, after 30- and 60-min reaction. More strikingly, Pt_1_Cu_2_/C presented significant activity (34% conversion) for tetraphenyl ethylene hydrogenation, in stark contrast to the negligible activity (<5%) of Pt_1_Cu_2_@MIL-101, in 60 min, because of the difficult substrate diffusion through MOF pore windows. Given the remarkable size-selective catalytic behavior of Pt_1_Cu_2_@MIL-101 and activity comparison with Pt_1_Cu_2_/C described above, the structure of MIL-101 should be well retained during loading MNPs. In addition, the results of size-selective catalysis once again demonstrate that the MNPs are mainly encapsulated inside MOF pores. 

## Discussion

In summary, we have successfully clarified the specific location of MNPs relative to MOF particles by several characterization techniques, including HAADF-STEM tomography with the slice technology, hyperpolarized ^129^Xe NMR spectroscopy, and positron annihilation spectroscopy (PAS). To the best of our knowledge, this is the first time to determine MNPs location relative to MOF particles as well as the pore structure information of MNPs/MOFs by ^129^Xe NMR and PAS techniques. The development of the current three reliable techniques would terminate the long-term controversy on the location of MNPs relative to MOFs, which remains to be a grand challenge and significantly affects the catalytic performance^47^. In addition, the obtained ultrafine PtCu alloy NPs are relatively low cost and exhibit superior catalytic performance to the monometallic counterparts owing to the synergetic effect between Pt and Cu, in the aerobic oxidation of primary alcohols using molecular O_2_ in pure water. The optimal Pt_1_Cu_2_@MIL-101 catalyst not only exhibits excellent catalytic activity and absolute selectivity to the corresponding aldehydes but also performs great recyclability by taking advantages of the confinement effect of MIL-101 for PtCu NPs even under moderately high reaction temperature and pressure. We believe that this work would pave the way to the detection of guest species location relative to diverse host porous materials and thus greatly promote the development of related host–guest nanocomposites for applications, especially in catalysis.

## Methods

### Materials and equipment

All chemicals were from commercial suppliers without further purification unless otherwise mentioned. Hexachloroplatinic acid hexahydrate (H_2_PtCl_6_·6H_2_O) was from Beijing Hwrk Chem Co., Ltd. Chromium(III) nitrate nonahydrate (Cr(NO_3_)_3_∙9H_2_O, 99%) was from Sigma-Aldrich. Powder X-ray diffraction patterns (PXRD) were conducted on a Japan Rigaku SmartLab^TM^ rotation anode X-ray diffractometer equipped or Holland X-Pert PRO fixed anode X-ray diffractometer equipped with graphite monochromatized Cu Kα radiation (*λ* = 1.54178 Å). The contents of Pt and Cu in the nanocomposites were quantified by an Optima 7300 DV inductively coupled plasma atomic emission spectrometer (ICP-AES). The X-ray photoelectron spectroscopy (XPS) measurements were carried out using an ESCALAB 250 high-performance electron spectrometer using monochromatized Al Kɑ (hν = 1486.7 eV) as the excitation source. The size, morphology, and microstructure of PtCu@MIL-101 samples were preliminarily investigated by using the transmission electron microscopy (TEM) and reconstructed slices from high-angle annular dark-field scanning transmission electron microscopy (HAADF-STEM) on JEOL-2010 and JEOL-2100F instruments with an electron acceleration energy of 200 kV. Nitrogen sorption isotherms were measured by using an automatic volumetric adsorption equipment (Micrometritics, ASAP 2020). Prior to nitrogen sorption measurements, the as-synthesized samples were sequentially activated in water, EtOH, and NH_4_Cl aqueous solution, finally dried in vacuum at 60 °C. Following that, the product was dried again by using the outgas function for 12 h at 150 °C. Catalytic reaction products were analyzed and identified by gas chromatography (GC, Shimadzu 2010 Plus with a 0.25 mm × 30 m Rtx^®^-5 capillary column).

### Tomography and slice techniques

To further verify whether NPs are really inside the MOF, a series of reconstructed slices were taken with 2° step as tilting sample stage from −62.6° to 62.6° by using FEI Tecnai G2 F20 TEM, operated at 200 keV. Computational reconstruction and visualization were conducted using the INSPECT3D and the AVIZO software package, respectively.

### Hyperpolarized ^129^Xe NMR measurements

The ^129^Xe NMR experiments were carried out at 110.6 MHz on the Varian Infinity-plus 400 spectrometer using a 7.5 mm probe. Prior to each experiment, the samples were subjected to dehydration at 423 K under vacuum (<10^−5^ Torr) for overnight. A flow of 1% Xe, 1% N_2_, and 98% He gas mixture was delivered at the rate of 150 cm^3^ min^−1^ to the sample in detection region via plastic tubing. Variable-temperature NMR measurements were performed in the range of 193–293 K. All one-dimensional spectra were acquired with 3.0 µs π/2 pulse, 100–200 scans, and 2 s recycle delay. The chemical shifts were referenced to the signal of xenon gas. Although this line was temperature dependent, its chemical shift variation would not be more than 1 ppm in the whole range of measurements because of the very low concentration of xenon.

### Positron annihilation measurement

The MIL-101, Pt@MIL-101, Pt_1_Cu_2_@MIL-101, and MOF-5 samples were pressed under a static pressure of about 5 MPa for about 3 min at room temperature to get disk shape pellets with a diameter of 10 mm and a thickness of 1 mm. Positron lifetime measurements were conducted using an ORTEC fast–fast coincidence system in nitrogen atmosphere. The time resolution of the system is about 240 ps in full width at half maximum (FWHM). Each spectrum was collected with a total count of 2 × 106. A 20 μCi source of ^22^Na was sandwiched between two identical sample pellets. Some notes should be pointed out with regards to the measurement: (i) the samples must be adequately dried overnight at 160 °C under vacuum and then should be always protected in nitrogen environment preventing from water absorption until test. (ii) Laminating the sample is a critical step. Generally, the sample should be pressed with a static pressure of about 5 MPa for about 3 min at room temperature to get disk shape pellets with a diameter of 10 mm (the two pieces must be kept equal mass and volume). (iii) The thickness of the sheet sample should be neither too thick nor too thin. In this work, 80 mg of sample with a thickness of 1 mm was just right. (iii) The entire tableting process should be rapid, and the samples were tested immediately after the pressing.

### Preparation of MIL-101

Typically, a mixture of 332 mg of terephthalic acid with 800 mg of Cr(NO_3_)_3_·9H_2_O in the presence of aqueous HF (0.4 mL, 2.0 mmol) and deionized water (9.5 mL) was reacted at 200 °C for 8 h. The as-synthesized MIL-101 was purified in water at reflux temperature for 24 h followed by in ethanol at 100 °C for 24 h for twice and washed with hot ethanol, and was further purified by NH_4_F solution. The result sample was finally dried overnight at 160 °C under vacuum for further use.

### Preparation of MOF-5

Typically, terephthalic acid (0.507 g) and triethylamine (0.85 mL) were dissolved in 40 mL of N,N-dimethylformamide (DMF); Zn(OAc)_2_·2H_2_O (1.7 g) was dissolved in DMF (50 mL). The Zn-based solution was added into the ligand solution under stirring over 15 min, forming a precipitate, and the mixture was kept stirring for over 2.5 h. The precipitate was purified by DMF, and then was separated by centrifugation followed by being immersed in CHCl_3_. The resultant MOF-5 powder was dried overnight at 120 °C under vacuum condition.

### Preparation of Pt@MIL-101, Cu@MIL-101, and PtCu@MIL-101

Typically, 200 mg of activated MIL-101 was suspended in 40 mL of a hydrophobic solvent of dry n-hexane, and the mixture was sonicated for around 20 min until it became homogeneous. After the mixture was stirred for a certain time, 0.30 mL of hydrophilic aqueous H_2_PtCl_6_·6H_2_O and/or Cu(NO_3_)_2_ solution with the desired concentration were added dropwise with a syringe pump over a period of 20 min with constant vigorous stirring. Subsequently, the resultant solution was continuously stirred for 3 h. The harvested sample was further dried followed by treating in a stream of 20% H_2_/Ar at 200 °C for 4 h to yield Pt@MIL-101, Cu@MIL-101, and PtCu@MIL-101. The Pt/Cu molar ratios were changed (2, 1, 0.5, 0.33, and 0.25) to optimize the activity of the resultant catalysts, while the total content of both metals was fixed to be 0.5 wt%. The successful preparation of ultrafine MNPs is affected by the suitable MIL-101 host, the metal precursor introduction technique (DSA) as well as the reduction method.

### Preparation of Pt_1_Cu_2_/MIL-101 and Pt_1_Cu_2_/C

Typically, 200 mg of activated MIL-101 powder or graphite carbon was dispersed in 20 mL of ultrapure water, and was subjected to ultrasonication for 20 min. A fresh H_2_PtCl_6_·6H_2_O and Cu(NO_3_)_2_ aqueous solution with the desired concentrations were added dropwise to the above solution under vigorous stirring for ~30 min. The suspension was then stirred at room temperature for another 24 h. The impregnated sample was washed with water adequately and was dried under vacuum at 60 °C for 24 h. The harvested sample was treated in a stream of 20% H_2_/Ar (40 mL min^−1^) at 200 °C for 4 h to yield Pt_1_Cu_2_/MIL-101 and Pt_1_Cu_2_/C.

### Catalytic performance evaluation for alcohol oxidation

The catalytic reaction was performed in a 10 mL Teflon-lined stainless-steel autoclave equipped with a pressure gauge and a magnetic stirrer. Upon drying at 120 °C under vacuum for 12 h, 100 mg of catalyst was dispersed in 5 mL of water containing 0.2 mmol of alcohol, and the mixture was sonicated for about 20 min until it became homogeneous. The vessel was then charged with O_2_ for ten times at room temperature, then pressurized with O_2_ to 5 bar for reaction. Subsequently, the reaction was conducted at 100 °C for 5 h with continuous stirring. After reaction, the catalyst was separated by centrifugation, thoroughly washing with ethanol, and then re-utilized in subsequent runs under identical reaction conditions. The yield of the product was analyzed by GC with an internal standard substance (dodecane).

### Catalytic performance evaluation for olefin hydrogenation

In a typical experiment, a mixture of catalyst (20 mg) and olefin (0.1 mmol) was ultrasonically dispersed in a mixture solvent (methanol and water, V:V = 5:1, 20 mL) or pure solvent (methanol, 20 mL) placed in round-bottomed flask (25 mL). The reduction started when hydrogen source (NH_3_BH_3_, 30 mg or NaBH_4_, 15 mg) was added into the flask. Catalytic yield of olefin hydrogenation reaction was identified by gas chromatography and ^1^H NMR.

## Supplementary information

Supplementary Information

Description of Additional Supplementary Files

Supplementary Movie 1

Supplementary Movie 2

Supplementary Movie 3

Supplementary Movie 4

## Data Availability

The data that support the findings of this study are available from the corresponding author upon reasonable request.
