# Supplementary material for: Location determination of metal nanoparticles relative to a metal-organic framework
Source: Nat Commun. 2019 Aug 1;10:3462. doi: 10.1038/s41467-019-11449-6 (PMC6671962; doi:10.1038/s41467-019-11449-6)
Supplement: Supplementary file 1 — Supplementary Information [file 41467_2019_11449_MOESM1_ESM.pdf]

Supplementary Information

Location determination of metal nanoparticles relative to a metal-organic framework

Yu-Zhen Chen[1,2], Bingchuan Gu[3], Takeyuki Uchida[4], Jiandang Liu[3], Xianchun Liu[5], Bang-Jiao Ye[3], Qiang Xu[4], & Hai-Long Jiang[1]*

[1] Hefei National Laboratory for Physical Sciences at the Microscale, CAS Key Laboratory of Soft Matter Chemistry, Department of Chemistry, Collaborative Innovation Center of Suzhou Nano Science and Technology, University of Science and Technology of China, Hefei, Anhui 230026, P. R. China [2] College of Chemistry and Chemical Engineering, Qingdao University, Qingdao, Shandong 266071, P. R. China [3] State Key Laboratory of Particle Detection and Electronics, University of Science and Technology of China, Hefei, Anhui 230026, P. R. China [4] National Institute of Advanced Industrial Science and Technology (AIST), Ikeda, Osaka 563-8577, Japan [5] State Key Laboratory of Catalysis, Dalian Institute of Chemical Physic, Chinese Academy of Sciences, Zhongshan Road 457, Dalian 116023, P. R. China.

*Correspondence and requests for materials should be addressed to H.-L.J (email: jianglab@ustc.edu.cn).

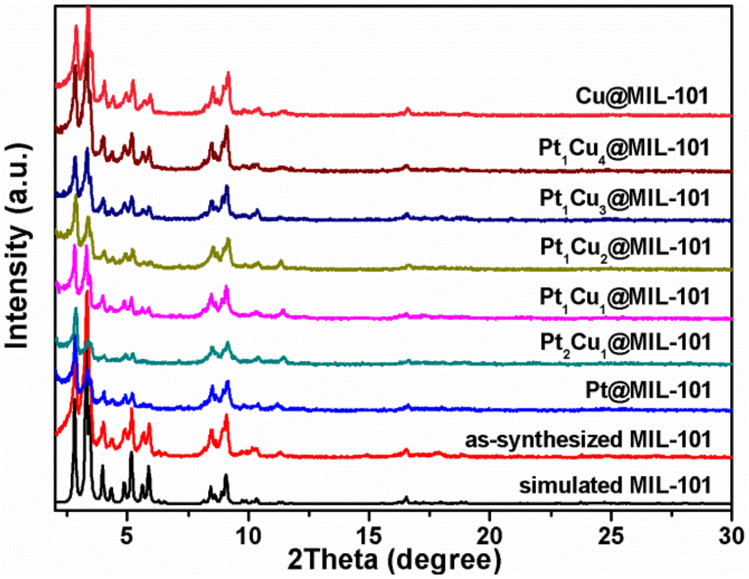

Supplementary Figure 1. PXRD profiles for simulated and as-synthesized MIL-101 as well as PtCu@MIL-101 catalysts.

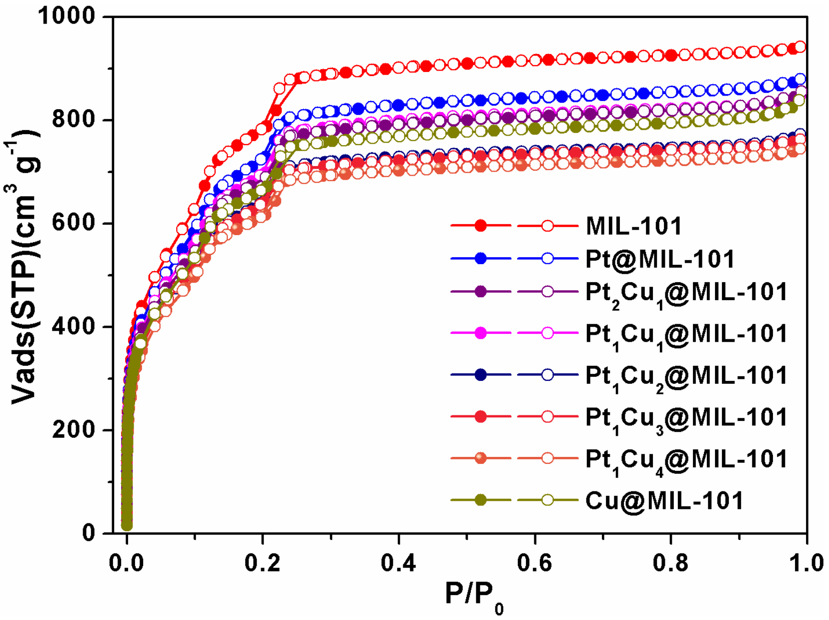

Supplementary Figure 2. N_2 sorption isotherms for different samples at 77 K. The BET surface areas are 2904, 2645, 2545, 2564, 2329, 2302, 2254 and 2479 m^2 g^{-1} for MIL-101, Pt@MIL-101, Pt_2Cu_1@MIL-101, Pt_1Cu_1@MIL-101, Pt_1Cu_2@MIL-101, Pt_1Cu_3@MIL-101, Pt_1Cu_4@MIL-101 and Cu@MIL-101, respectively. The MIL-101 used in all samples are from the same batch.

Supplementary Table 1. Inductively coupled plasma-atomic emission spectrometry (ICP-AES) data for PtCu@MIL-101 catalysts.

Nanoparticles	(Pt + Cu) content (%)	n_{Pt}/n_{Cu} (mol/mol)
Pt	0.52	-
Pt_2Cu_1	0.41	2.3/1
Pt_1Cu_2	0.34	1/2.1
Pt_1Cu_3	0.49	1/3.3
Pt_1Cu_4	0.49	1/4.5
Cu	0.64	-

The results have confirmed that the actual contents of Pt and Cu are close to the nominal values and the Pt/Cu ratios match the predesigned trend.

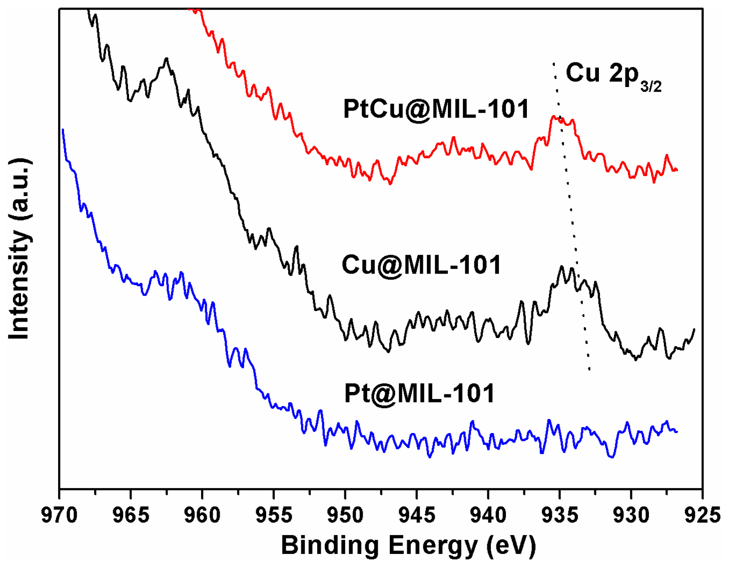

Supplementary Figure 3. XPS spectra of Cu 2*p* for Pt@MIL-101, Cu@MIL-101 and PtCu@MIL-101.

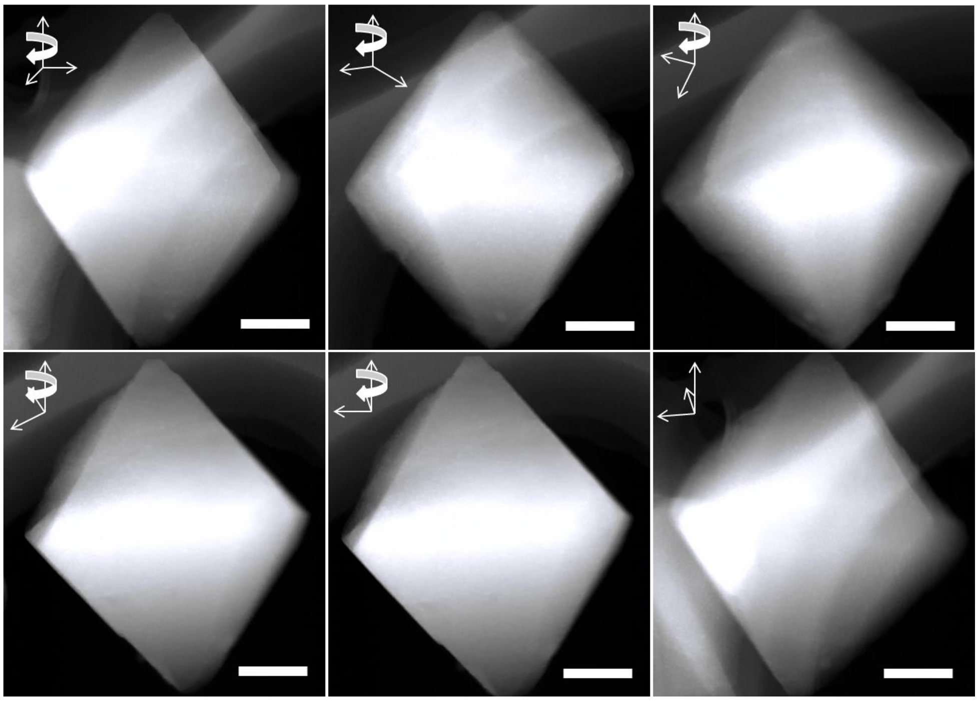

Supplementary Figure 4. The representative HAADF-STEM images captured from the video with a series of tilting angles for Pt@MIL-101 sample taken with 2 ° tilt increment step from -62.6 ° to 62.6 °. Alpha tilt axis is parallel with Z direction shown on the images. The scale bar on the images is 100 nm.

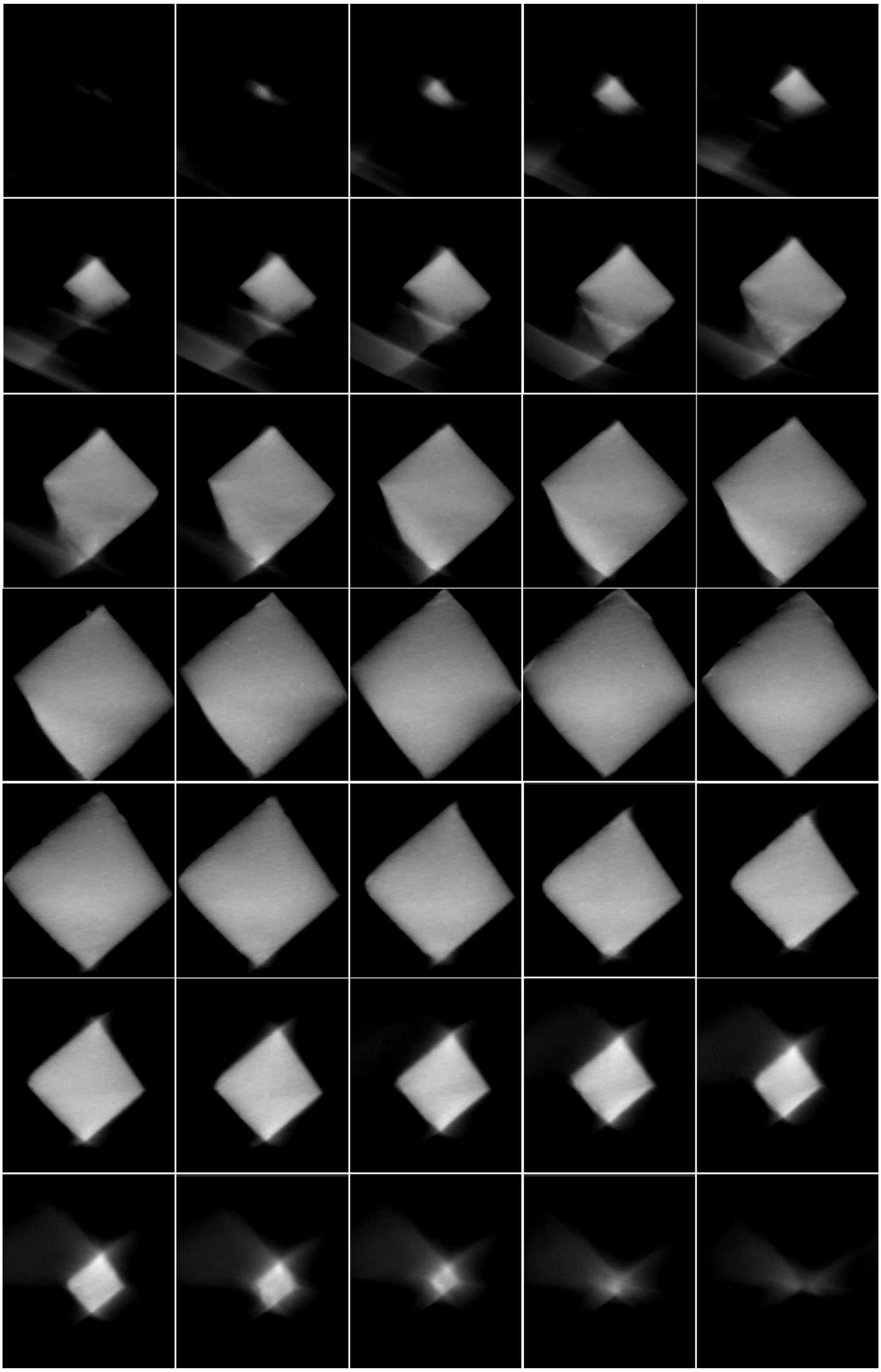

Supplementary Figure 5. The reconstructed slice images throughout the entire Pt@MIL-101 skeleton, revealing that Pt NPs (bright spots) are highly dispersed and embedded inside the MOF particle.

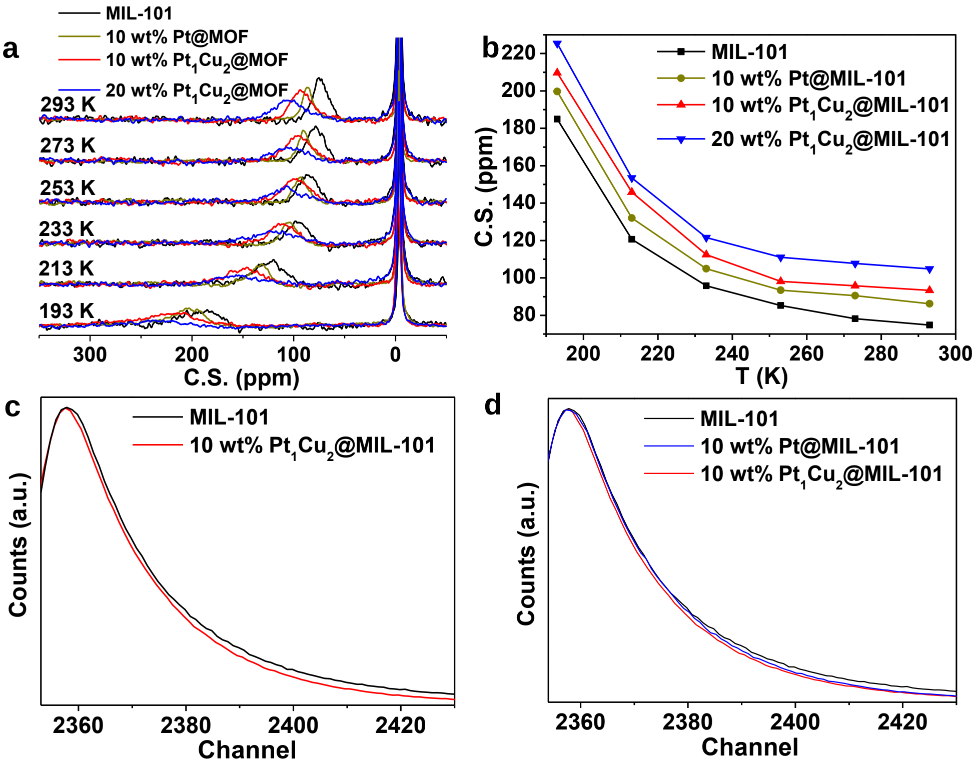

Supplementary Figure 6. a, Temperature-dependent hyperpolarized ^{129}Xe NMR spectra for MIL-101, 10 wt% Pt@MIL-101, 10 wt% and 20 wt% PtCu@MIL-101. The measurements were performed in the range of 193-293 K. **b**, The ^{129}Xe chemical shift difference for MIL-101, 10 wt% Pt@MIL-101, 10 wt% and 20 wt% Pt_1Cu_2@MIL-101 at different temperatures. **c**, Positron lifetime spectra for MIL-101 and 10 wt% Pt_1Cu_2@MIL-101. **d**, Positron lifetime spectra for MIL-101, 10 wt% Pt@MIL-101 and 10 wt% Pt_1Cu_2@MIL-101.

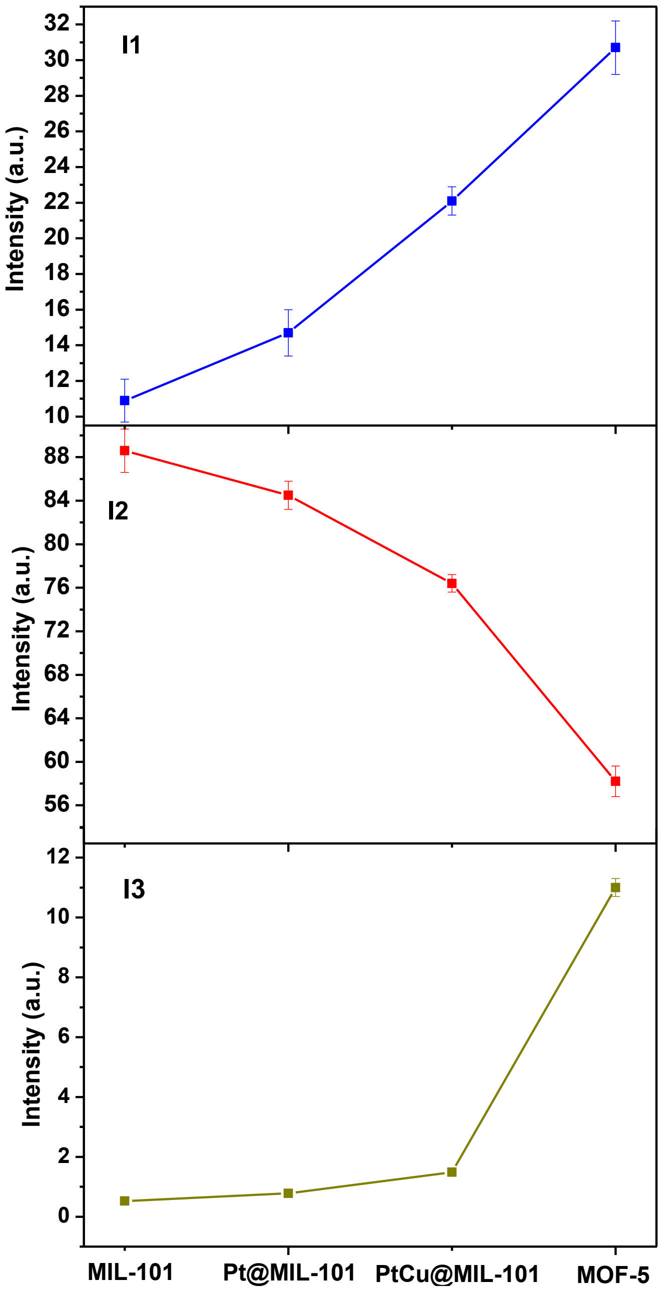

Supplementary Figure 7. The intensities of I1, I2 and I3 with error for MIL-101, Pt@MIL-101, PtCu@MIL-101 and MOF-5. The error-bars are standard errors of the mean.

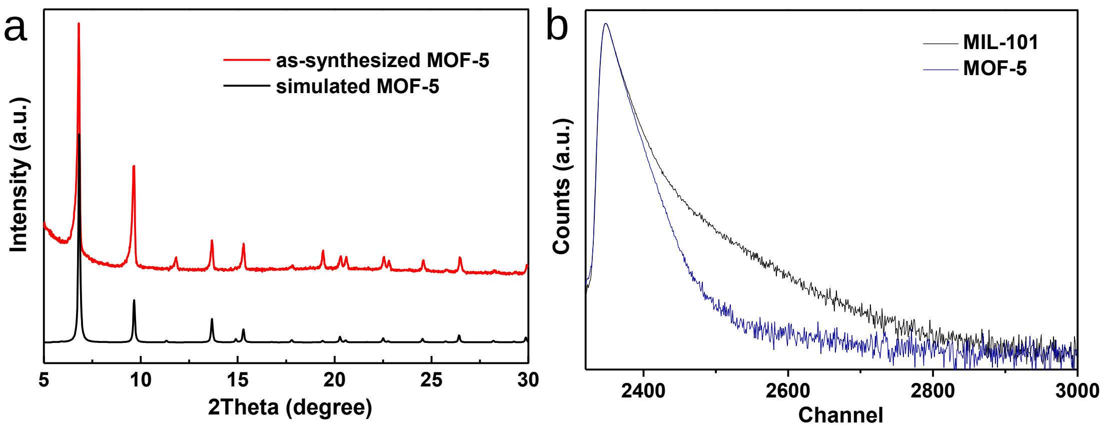

Supplementary Figure 8. a, PXRD profiles for simulated and as-synthesized MOF-5 and **b**, Positron lifetime spectra for MOF-5 and MIL-101 based on positron annihilation experiments.

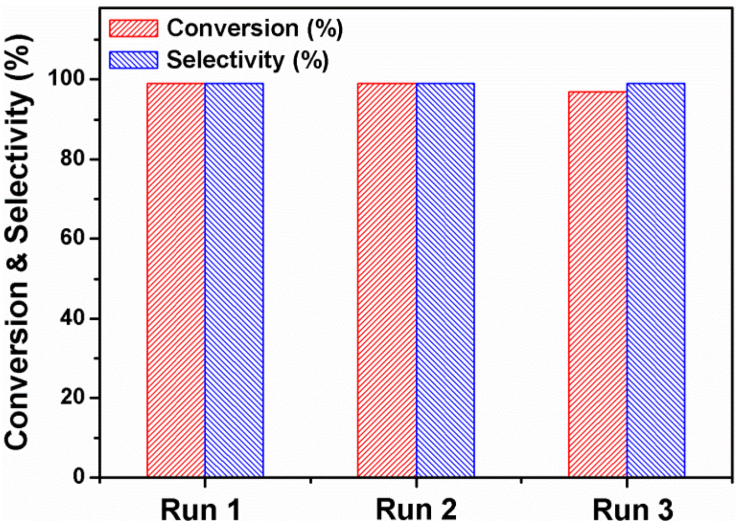

Supplementary Figure 9. Recycling performance (the conversion of benzyl alcohol and selectivity toward benzaldehyde) over Pt_1Cu_2@MIL-101. Reaction conditions: catalyst (100 mg), alcohol (0.2 mmol), H_2O (5 mL), O_2 (0.5 MPa), 373 K, reaction time for each run: 5 h.

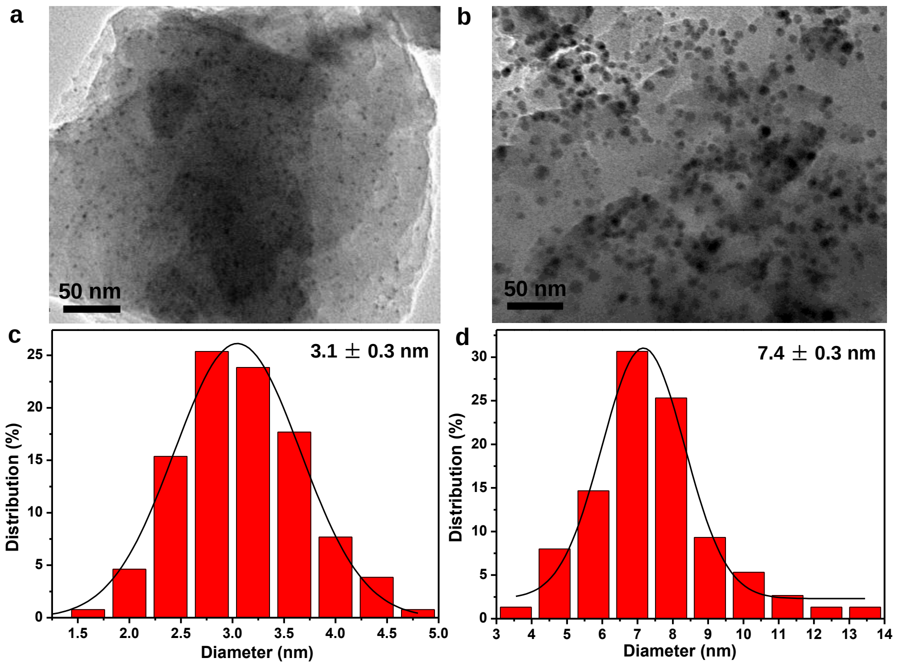

Supplementary Figure 10. **a**, The TEM image and **c**, the corresponding PtCu size distribution of Pt_1Cu_2/MIL-101 before reaction. **b**, The TEM image and **d**, the corresponding PtCu size distribution of Pt_1Cu_2/MIL-101 after three catalytic cycles.

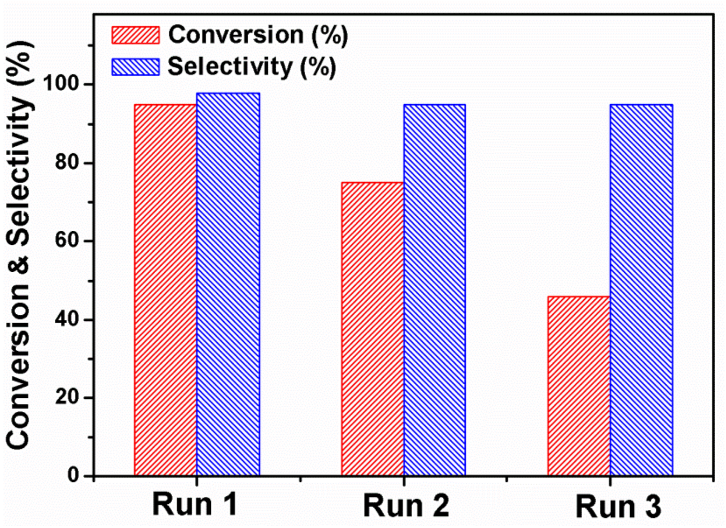

Supplementary Figure 11. Recycling performance (the conversion of benzyl alcohol and selectivity toward benzaldehyde) over Pt_1Cu_2/MIL-101. Reaction conditions: catalyst (100 mg), alcohol (0.2 mmol), H_2O (5 mL), O_2 (0.5 MPa), 373 K, reaction time for each run: 5 h.

Supplementary Table 2. Catalytic performance of Pt_1Cu_2@MIL-101 and Pt_1Cu_2/C for hydrogenation reactions.[a]

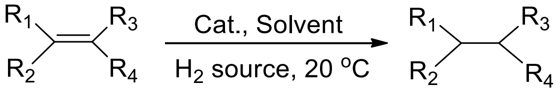

Entry	Substrate	Yield (%)[b] Pt_1Cu_2@MIL-101	Yield (%)[b] Pt_1Cu_2/C	Time (min)
1[c]	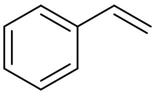	>99	>99	10
2[c]	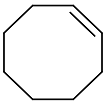	29	56	30
		70	>99	60
3[d]	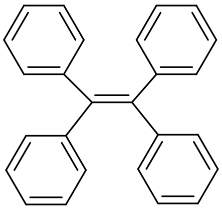	<3	~34	60

[a]Reaction conditions: catalyst (20 mg), olefin (0.1 mmol), 293 K, reaction time is 1 h. [b]Yield was analyzed by GC and ^1H NMR, and *n*-dodecane was used as the internal standard for GC. [c]MeOH/H_2O (20 mL), NH_3BH_3 (30 mg). [d]Solvent (ethanol, 20 mL) and hydrogen source ($NaBH_4$, 15 mg).

Supplementary Movie files:

HAADF-STEM and tomographic slices videos for Pt@MIL-101 and Pt_1Cu_2@MIL-101.

Video 1: 3D HAADF-STEM at consecutive tilt angles from -62.6 ° to 62.6 ° with each 2 ° tilt increment for Pt@MIL-101.

Video 2: 3D HAADF-STEM at consecutive tilt angles from -62.6 ° to 62.6 ° with each 2 ° tilt increment for Pt_1Cu_2@MIL-101.

Video 3: Tomographic slices of Pt_1Cu_2@MIL-101.

Video 4: Tomographic slices of Pt@MIL-101.
