# Supplementary material for: Location determination of metal nanoparticles relative to a metal-organic framework
Source: Nat Commun. 2019 Aug 1;10:3462. doi: 10.1038/s41467-019-11449-6 (PMC6671962; doi:10.1038/s41467-019-11449-6)
Supplement: Supplementary file 2 — Description of Additional Supplementary Files [file 41467_2019_11449_MOESM2_ESM.docx]

**Description of Additional Supplementary Files**

File Name: Supplementary Movie 1
Description: 3D HAADF-STEM at consecutive tilt angles from -62.6° to 62.6° with each 2° tilt increment for Pt@MIL-101

File Name: Supplementary Movie 2
Description: 3D HAADF-STEM at consecutive tilt angles from -62.6° to 62.6° with each 2° tilt increment for Pt1Cu2@MIL-101

File Name: Supplementary Movie 3
Description: Tomographic slices of Pt1Cu2@MIL-101

File Name: Supplementary Movie 4
Description: Tomographic slices of Pt@MIL-101.
